# Methadone-Induced Toxic Brain Damage 

**DOI:** 10.1155/2013/602981

**Published:** 2013-05-22

**Authors:** Jérôme Corré, Jérôme Pillot, Gilles Hilbert

**Affiliations:** Medical Intensive Care Unit, Bordeaux University Hospital, Pellegrin Hospital, 33000 Bordeaux, France

## Abstract

A 29-year-old man presented with comatose after methadone intoxication. Cerebral tomography only showed cortico-subcortical hypodense signal in the right cerebellar hemisphere. Brain MRI showed a rare imaging of FLAIR and DWI hyperintensities in the two cerebellar hemispheres as well as basal ganglia (globi pallidi), compatible with methadone overdose. To our knowledge this is the first reported case of both cerebellar and basal ganglia involvement in methadone overdose.

## 1. Case Report

A 29-year-old male was found comatose, 14 hours after at least sixty milligrams of witnessed methadone ingestion during a meeting of drug addicts.

His main medical history revealed drugs addiction such as methadone, cannabis, alcohol, and cyamemazine.

The first medical examination showed a patient in a deep coma (Glasgow Coma Scale score of 3), with weakly reactive intermediate pupils, central temperature of 35.3°C, low blood arterial pressure (BP 70/50 mmHg), bradypnea at 8 per min, and an oxygen saturation at 88%. Capillary blood glucose was normal at 132 mg·dL^−1^. After orotracheal intubation and intravenous fluid resuscitation, the patient was transferred into our medical Intensive Care Unit. On arrival, he was in hemodynamic instability (BP 60/40 mmHg), with 70/min pulse and threatening hyperkalemia combining atrio-ventricular block I (PR 520 ms), wide QRS to 160 ms with an aspect of atypical right bundle branch block, and wide T waves on ECG. His neurological examination noted a global hypotonia, abolition of tendon reflexes, and no head trauma. Biology confirmed severe hyperkalemia (9.4 mmol·L^−1^) with acute renal failure (creatinine 2.6 mg·dL^−1^) due to an uncommon left gluteal crush syndrome after prolonged immobilization. Serum CPK level was very high at 148,700 UI·L^−1^ on day 1. Blood analysis was positive for alcohol, cannabis, methadone (146 ng·mL^−1^), and benzodiazepines. Qualitative serum assays were negative for tricyclic antidepressants, amphetamines, barbiturates, opiates, cocaine, and carbamates.

Hyperkalemia was treated with calcium gluconate, sodium bicarbonate, and haemodialysis, and the patient received vasopressor support with norepinephrine after fluid challenge.

Computerized tomography of the brain pointed out a cortico-subcortical hypodense signal in the right cerebellar hemisphere.

**Figure 1:**
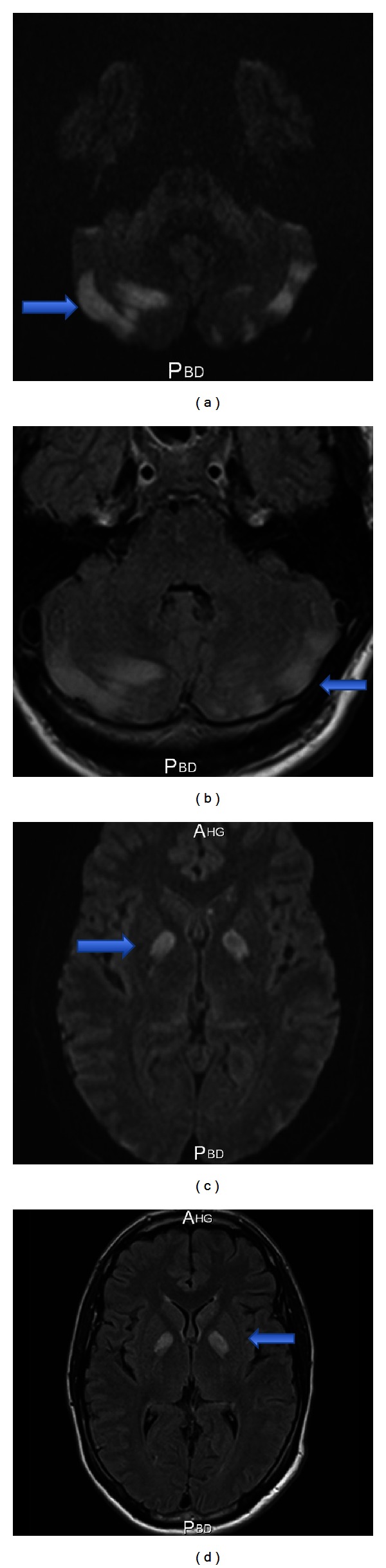
Cerebral IRM showing FLAIR (on the left side) and DWI (on the right) hyperintensities in both cerebellar hemispheres (panels (a)-(b)) and basal ganglia (panels (c)-(d)).

Brain magnetic resonance imaging (MRI) showed FLAIR (Figures 1(a) and 1(c)) and DWI (Figures 1(b) and 1(d)) hyperintensities in the two cerebellar hemispheres and basal ganglia (globi pallidi), compatible with methadone overdose. The angio-MRI found no damage of the supra-aortic trunks.

Finally, the patient had a good recovery, except persistent renal failure and kinetic cerebellar syndrome.

## 2. Discussion 

In this case, purely ischemic lesions on the brain resulting from cerebral hypoperfusion could be discussed; nevertheless both the symmetry and the combination of the two injured regions (cerebellum and globus pallidus) are strongly suggestive of toxic damage, such as those described in the literature with heroin and oxycodone. Methadone is a synthetic opioid, with high affinity for the mu class of opioid receptors, which predominate in the cerebellum and limbic systems in humans [1]. Several reported cases illustrate the cerebellarpredominant toxicity of methadone and heroin overdose [2, 3]. Few cases also show a notable involvement of the basal ganglia [4]. To our knowledge this patient is the first reported case of cerebellar and basal ganglia involvement in methadone overdose.
